# Supplementary material for: Learning curve and surgical outcome of robotic assisted colorectal surgery with ERAS program
Source: Sci Rep. 2022 Nov 29;12:20566. doi: 10.1038/s41598-022-24665-w (PMC9709162; doi:10.1038/s41598-022-24665-w)
Supplement: Supplementary file 1 — Supplementary Information 1. [file 41598_2022_24665_MOESM1_ESM.zip › LSC Raw data-Ñ[▒K/078/O_ñw╝╨░O▒Kñσ.pdf]

正式摘要(電子簽章：0000001895334132)

請選擇...reporter

臺中榮民總醫院

## Operation Note

[redacted]

- 手術日期：2020/04/24
- 手術醫師：蔣鋒帆
- Anesthesia：GE

## Diagnosis

- Pre-operative diagnosis : Rectal tumor
- Post-operative diagnosis : Rectal cancer, r/o lung mets, s/p laparoscopic LAR on 20200424, cT3N0M1a

## Surgical Indication

- Rectal tumor bleeding with anemia status

## Operation

- 1. Laparoscopic LAR with end-to-end anastomosis, double stapling, endo GIA 60/4.1mm, CDH 29mm. Re-inforcement of silk: nil, air tight test : done
  2. Bilateral donuts: intact
  3. Re-peritonealization : No
  4. Takedown of splenic flexure: No
  5. Proximal margin: 15cm, distal margin: 2.5cm
  6. Vessel ligation: IMA above left colic artery
  7. 19 Fr. hemovac drain x 1 at the pelvis

## Finding

- 1. An borrmann type II ulcerative tumor, about 3*2 cm in size, located at upper rectum, 10cm FAV, which invaded to the pericolic tissue. No palpable lymph nodes at IMA root or mesorectum. Distal and proximal of tumor was tattooed. Clinical stage: T3N0M1a
  2. No visible peritoneal seeding, no liver mets was found.
  3. Omentum adhesion to D-colon and upper rectum due to L-P shunt catheter. Adhesion between T-colon and D-colon, s/p enterolysis.

## Procedure

- Under general anesthesia, the patient was placed in Trendelenburg lithotomy position. The abdomen and perineum was prepped and draped in standard sterile fashion. A small incision was made near umbilicus and varess needle was inserted. Pneumoperitoneum was established with CO2 inflation. A 10mm trochar was inserted below umbilicus. Laparoscopy was inserted and then another 5mm and 10mm trochars were inserted over RUQ and RLQ respectively under laparoscopic guide. Another 5mm trochar was inserted at LLQ under laparoscopic guide. Mesosigmoid colon was hold by grasper and dissection was made to separate mesocolon from retroperitoneum and further dissection was done toward mesorectum. IMA root, IMA, l't cloic artery, S1 artery were identified and endoclips were applied below l't colic artery. IMV was also divided after endoclips applied. Dissection was done above the Gerota's fascia and toward the peritoneal attachments. The colon was mobilized from retroperitoneum overlying the sacral promontory and into the pelvis. The incision was extended laterally and medially across the peritoneal reflection. The mesorectum was then mobilized. Distal descending colon and middle rectum were mobilized. The rectum was divided with endo-GIA 60/4.1mm. Laparoscopy and umbilicus trochars were removed. A small midline incision was made at umbilical region. The rectum was pulled out and looped by gauze. The proximal limb was pulled out and dissection was done at the selected site of proximal cut end with ligation of marginal artery. Purse-string clamp was applied and the specimen was removed. End-to-end

- anastomosis was made by CDH 29mm. One 19Fr. hemovac drainage was placed at pelvis. The midline wound was closed with No.1 Vicryl suture. The wound was closed by dermabond. Under stable condition, the patient was sent to POR.
- Estimated blood loss: minimum
- 器械數量: 正確
- 紗布數量: 正確

## Image

- PAINT
  - colon & rectum

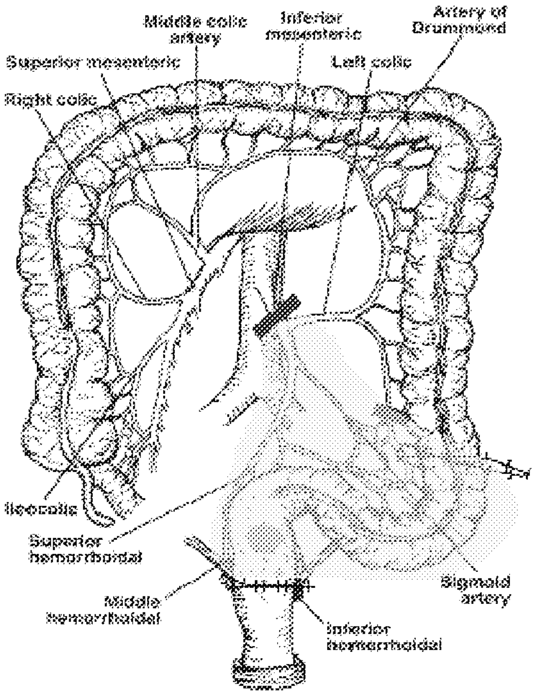


  - abdomen

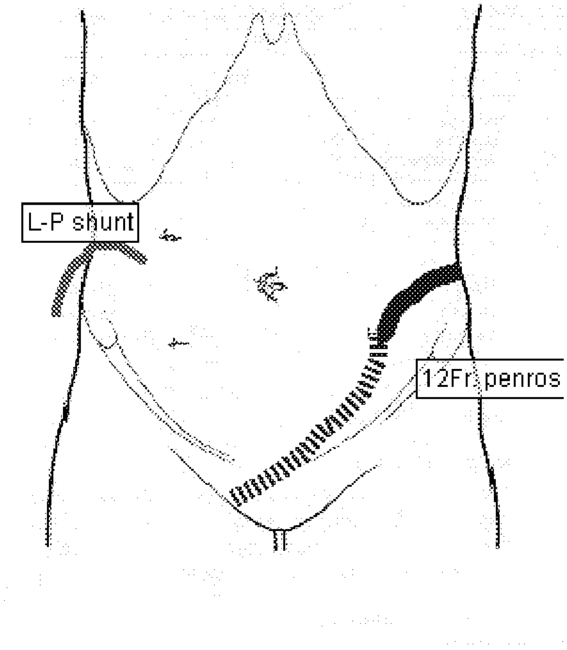


編輯主任 2062F蔣鋒帆、主治 2320B林昌霖

正式摘要(電子簽章：**000000189534132)**

| | | | | | |
|---|---|---|---|---|---|
| | | 請選擇...reporter | | | |

doctorRank:B
